# Enhanced Two-Step Virtual Catchment Area (E2SVCA) model to measure telehealth accessibility

**DOI:** 10.1007/s43762-023-00092-z

**Published:** 2023-04-03

**Authors:** Yaxiong Shao, Wei Luo

**Affiliations:** Department of Earth, Atmosphere and Environment, Northern Illinois University, 1425 W. Lincoln Hwy, DeKalb, IL 60115 USA

**Keywords:** Healthcare equality, Telehealth care accessibility, Public health, E2SFCA, 2SVCA, E2SVCA, COVID-19

## Abstract

The use of telehealth has increased significantly over the last decade and has become even more popular and essential during the COVID-19 pandemic due to social distancing requirements. Telehealth has many advantages including potentially improving access to healthcare in rural areas and achieving healthcare equality. However, there is still limited research in the literature on how to accurately evaluate telehealth accessibility. Here we present the Enhanced Two-Step Virtual Catchment Area (E2SVCA) model, which replaces the binary broadband strength joint function of the previous Two-Step Virtual Catchment Area (2SVCA) with a step-wise function that more accurately reflects the requirements of telehealth video conferencing. We also examined different metrics for representing broadband speed at the Census Block level and compared the results of 2SVCA and E2VCA. Our study suggests that using the minimum available Internet speed in a Census Block can reveal the worst-case scenario of telehealth care accessibility. On the other hand, using the maximum of the most frequent available speeds reveals optimal accessibility, while the minimum of the most frequent reflects a more common case. All three indicators showed that the 2SVCA model generally overestimates accessibility results. The E2SVCA model addresses this limitation of the 2SVCA model, more accurately reflects reality, and more appropriately reveals low accessibility regions. This new method can help policymakers in making better decisions about healthcare resource allocations aiming to improve healthcare equality and patient outcomes.

## Introduction

Telehealth refers to the use of electronic information and telecommunications technologies to support and promote long-distance clinical health care, patient and professional health-related education, and public health and health administration (U.S. Department of Health & Human Services, 2020). Telehealth has been in existence since at least the 1960s (e.g., Darkins & Cary, 2000) and has many advantages, including convenience, reaching people with mobility limitations, and improving access in rural area. We have witnessed the increase in telehealth usage in recent decades, particularly during the COVID-19 pandemic. According to findings reported by Ng and Park (2021), 81.2% of survey respondents reported that their medical providers offered telehealth services during the pandemic. Another study suggested that telehealth was rapidly used by doctors and patients to reduce COVID-19 transmission (Maleka & Matli, 2022). Telehealth also offered significant help to those with chronic diseases that required seeing a doctor regularly to understand and manage their conditions (Bitar & Alismail, 2021). Marks et al., (2022) concluded that the increased usage of telehealth improved healthcare accessibility. Furthermore, a recent study found that telehealth will continue to be widely used after the pandemic as more than 73% of patients expect to obtain care via telehealth after the pandemic (Doximity, 2022). The impact of telehealth can go beyond the pandemic, especially for primary health care (Beheshti et al., 2022).

The growing popularity of telehealth during the pandemic have prompted researchers to pay more attention to healthcare accessibility evaluation. Previous studies have largely focused on measuring healthcare accessibility for traditional physical healthcare services, using methods such as the Two-Step Floating Catchment Area (2SFCA) method (Luo & Wang, 2003), the Enhanced Two-Step Floating Catchment Area (E2SFCA) method (Luo & Qi, 2009), Modified Two-Step Floating Catchment Area (M2SFCA) method (Delamater, 2013), Variable Catchment Sizes for the Two-Step Floating Catchment Area (V2SFCA) (Luo & Whippo, 2012), Rational Agent Access Model (RAAM) (Saxon & Snow, 2020), and others. However, recent research suggested that telehealth can significantly improve health care access in rural areas (Lum et al., 2020; Nagata, 2021; Walter Panzirer, 2021). Some studies concluded that even though telehealth delivery had improved accessibility for those living in rural areas, the digital divide still prevented the elderly, those with lower incomes, and racial/ethnic minority groups from receiving health care services (Cortelyou-Ward et al., 2020; Saeed, 2021). Therefore, it is critical to accurately assess the accessibility of telehealth. Alford-Teaster et al. (2021) developed the Two-Step Virtual Catchment Area (2SVCA) method which incorporates FCC broadband speed data into the 2SFCA method to measure access to telehealth care. However, the 2SVCA model only considers two scenarios: having access to the Internet and not having access to the Internet. This binary function does not account for all cases in the real world. For instance, audio issues are the most common difficulty for patients receiving telehealth services, accounting for 26% of all cases according to the 2020 U.S. Telehealth Satisfaction Survey (J.D. Power, 2020). Even though these groups of the population already have access to the Internet, the quality of the Internet may not be sufficient to meet the requirements of telehealth platforms. To address these shortcomings, our Enhanced Two-Step Virtual Catchment Area (E2SVCA) method adopts a step-wise function that divides the broadband speed into several categories and assigns the weights to different categories as E2SFCA did.

## Study area and data

We selected Cook County as the location for our study due to its inclusion of the third largest city in the United States, Chicago. Cook County is an urban county situated in the upper northeastern region of Illinois, with over 800 local governmental units within its boundaries and a population of approximately 5.2 million individuals, making it the second most populated county in the country (Cook County Government, n.d.). More than 40 percent of the population of Illinois (12.84 million) lives within Cook County.

**Fig. 1:**
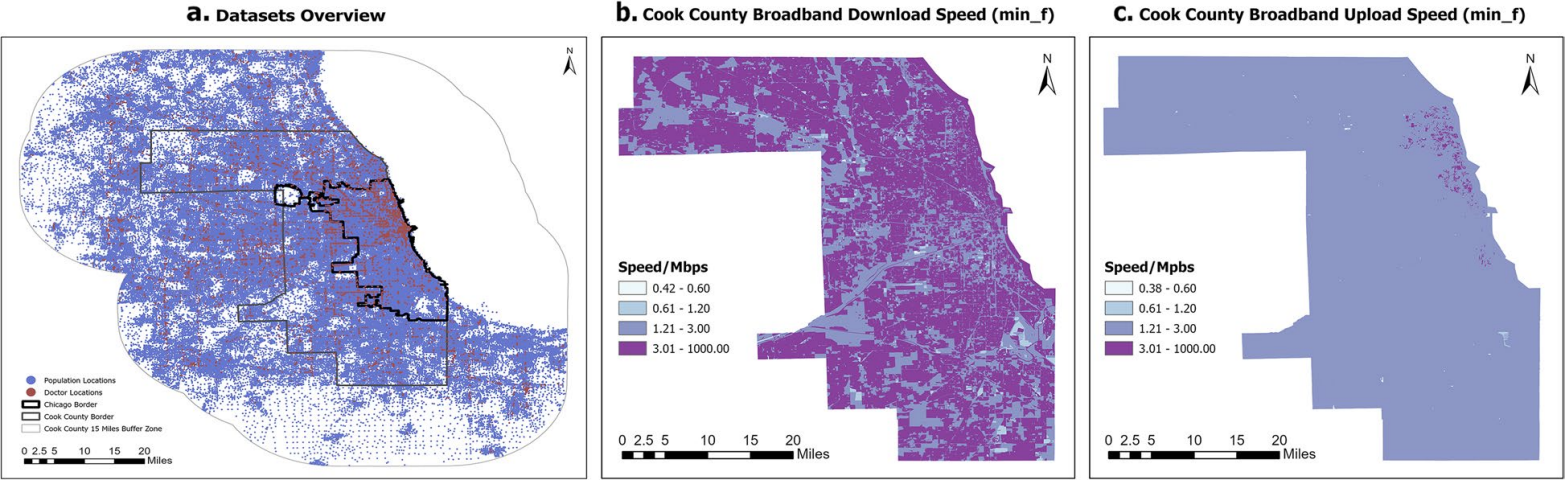
Study area and data

To prevent the edge effect from influencing our study, we extended our data to a 15-mile buffer zone around Cook County (Fig. 1a). However, we only report findings within the County. Here is a brief overview of the datasets we utilized in this study:

- (1) Doctors data: We crawled the data of 29,513 doctors from sharecare.com using a distributed web crawling spider based on the Scrapy framework. This dataset was aggregated into the U.S. Census Block level, with the location of doctors represented by the census block centroids. This dataset covers 5018 Census Blocks in the study area (including the buffer zone), with 20,456 doctors located within Cook County and distributed across 3,158 Census Blocks (Sharecare, 2020).
- (2) Boundaries and population data: We extracted the 2010 Census Block boundary data and population data from the U.S. Census Bureau’s website. The study area comprises 166,980 Census Blocks and 8,377,745 individuals, of which 99,036 Census Blocks and 5,194,675 individuals are located within Cook County. We selected Census Block level data because it is the finest geographic level available.
- (3) Road networks: We downloaded the U.S. Midwest OpenStreetMap road networks from Geofabrik.de, which were processed using the Open Source Routing Machine (OSRM) and used to calculate the OD matrix from each Census Block centroid of the population to each Census Block of doctors (Luxen & Vetter, 2011). We chose OpenStreetMap as our road network data source because it provides free and open source data that is frequently updated. Additionally, a previous study has shown that OpenStreetMap data is highly consistent with other online road network providers, such as Google Maps, and ArcGIS Online (Delmelle et al., 2019).
- (4) Federal Communications Commission (FCC) broadband speed dataset: We obtained this dataset from FCC websites, which provides broadband speed (download and upload) for each service provider at the U.S. Census Block level (Federal Communications Commission, 2022). Download speed refers to the rate at which data is transferred from the server to the user’s device, while upload speed refers to the rate at which data is transferred from the user’s device to the server. The primary difference between these two speeds is the direction of data transfer (Supan, 2023). For instance, for a 1 to 1 1080p video conference on Zoom, a minimum 3.8Mbps upload speed and 3.0Mbps download speed are required. Figure 1b and c illustrate the minimum of the most frequently available download and upload broadband speeds for Cook County. More details can be found in Section 4.2.
- (5) Zoom Internet bandwidth requirements: As Zoom is the most widely used video call application globally, which also provides specific bandwidth requirements for making video calls on their website. We used the Internet speed requirements based on a 1 to 1 Zoom video call bandwidth, with high-quality video requiring 600kbps (up/down), 720p HD video requiring1.2Mbps (up/down), and 1080p HD video requiring 3.8Mbps/3.0Mbps (up/down). To simplify the calculation, we assumed that both upload and download requirements for 1080p HD video were the same (3.0Mbps). This data was used to determine the weights of the broadband (Zoom Support, 2022). More details can be found in Section 3.2.

## Methodology and data processing

### Methodology

The traditional two-step floating catchment area (2SFCA) method and its variations have become popular in modeling spatial accessibility to healthcare. To address the issue of uniform access within the catchment, the enhanced two-step floating catchment area (E2SFCA) method was developed, which incorporates a distance decay function into the 2SFCA model. The formula for this approach can be expressed as follows:

1 $$A_i=\sum\limits_{j=1}^n\left[\frac{S_jf\left(d_{ij}\right)}{\sum_{k=1}^m\left(D_kf\left(d_{kj}\right)\right)}\right]$$

where ${A}_{i}$ is the accessibility at location$i$, and ${S}_{j}$ is the supply at location $j$; ${D}_{k}$ is the demand at location$k$; $f$*is* the distance decay function between the supply and demand locations.${d}_{i j}$, ${d}_{k j}$ denote the distance or travel time between demand location $i$ and supply location$j$, or demand location $k$ and supply location$j$, respectively.

Equation (1) can be explained as follows:

First, for each supply location $j$, search all demand locations $k$ that are within the catchment, and compute the supply–demand-ratio ${R}_{j}$, with demand discounted by the distance decay function $f\left({d}_{k j}\right)$:

2 $$R_j=\frac{S_j}{\sum_{k=1}^m\left(D_k\;f\left(d_{k\;j}\right)\right)}$$

Second, for each demand location $i$, search all supply locations $j$ within the catchment, and summing up the supply–demand-ratio ${R}_{j}$ at those locations, discounted by the decay function $f\left({d}_{i j}\right)$, to obtain the accessibility ${A}_{i}$ at location $i$:

3 $$A_i=\sum\limits_{j=1}^nR_j\;f\left(d_{i\;j}\right)$$

The two-step virtual catchment area method is a novel approach for assessing telehealth accessibility (Alford-Teaster et al., 2021). It replaces the distance decay function in the E2SFCA (Eq. (1)) with a broadband strength joint function that considers both upload and download speeds. The formula for virtual accessibility can be expressed as Eq. (4):

4 $$VA_i=\sum\limits_{j\in\left(d_{ij}\leq d_0\right)}^n\left[\frac{S_if(b_ib_j)}{\sum_{k\in\left(d_{ki}\leq d_0\right)}^m\left(D_kf\left(b_kb_i\right)\right)}\right]$$

where ${VA}_{i}$ is the virtual accessibility at location $i$, $f({b}_{i}{b}_{j})$ is the broadband strengths joint function for broadband qualities. It is important to note that in this case, the catchment is determined by travel time on the road network since telehealth often times serves as a supplement to the in-person healthcare services and would not fully replace the traditional in-person visits; most people still prefer seeking care in nearby areas (often with the same doctors) even when using telehealth (Alford-Teaster et al., 2021). However, Alford-Teaster et al. (2021) used a binary function $f({b}_{i}{b}_{j})=1$ or $f({b}_{i}{b}_{j}) =0$ to assess the telehealth care accessibility. This function equals 1 if both supply and demand have Internet access, indicating that the telehealth care services can be delivered. Conversely, if either the demand or the supply side lacks access to the Internet, the broadband communication strength function's value will be 0, and no telehealth service can be provided. However, this approach neglects an important variable, namely, the required broadband speed for video calls. For instance, assume that a video call requires minimum Internet speed of 10Mbps. If the patient has only 1Mbps Internet speed, and the telehealth care provider has 100Mbps. In this case, $f({b}_{i}{b}_{j})=1$, indicating that telehealth services will be provided even if a video conference cannot be established due to insufficient network speed. In our study area, all Census Block have at least one Internet service provider. Applying the 2SVCA model, the joint function for broadband strengths would yield a value of 1 for all cases, simplifying the model to the traditional 2SFCA model, which does not consider distance decay or broadband speed.

To overcome these limitations, we propose categorizing bandwidth requirements and using the minimum upload and download speeds of both patients and doctors as the determining factor for two-way communication in telehealth care. We present a step-wise broadband strengths joint function in Eq. (5) and call this model the Enhanced Two-Step Virtual Catchment Area (E2SVCA) method:

5 $$f(b_{iu},b_{id}{,b}_{ju}{,b}_{jd})=\left\{\begin{array}{cc}w_0&0\leq x<x_1\\\cdots&\cdots\\w_n&x_n\leq x<x_{max}\end{array}\right.$$

where $x=min({b}_{iu},{b}_{id}{,b}_{ju}{,b}_{jd})$, ${b}_{iu}$ represents the Internet upload speed at location $i$ and ${b}_{id}$ is the download speed at location$i$, ${b}_{ju}$ is the upload speed at location $j$, ${b}_{jd}$ is the download speed at location$j$. ${w}_{0},\cdots , {w}_{n}$ are weights range from 0 and 1, while ${x}_{1},\cdots , {x}_{n}$ represent bandwidth requirements for different quality video conferences. In our study, we can express the joint function of broadband strengths as Eq. (6), which is based on the Zoom bandwidth requirements listed in Table 1:

**Table 1:** Weights based on zoom bandwidth requirement

Speed/Mbps	Weight
0 ~ 0.6	0
0.6 ~ 1.2	0.33
1.2 ~ 3.0	0.66
3.0 ~ 9999.0	1

6 $$f(b_{iu},b_{id}{,b}_{ju}{,b}_{jd})=\left\{\begin{array}{cc}0&0\;\leq x<0.6\\0.33&0.6\leq x<1.2\\0.66&1.2x\leq x<3.0\\1&3.0x\leq x<9999.0\end{array}\right.$$

After obtaining the weight function based on Internet speed, we can calculate the telehealth accessibility by applying it to Eq. (4). The resulting equation, Eq. (7), is as follows:

7 $$V{A}_{i}=\sum\limits_{j\in \left({d}_{ij}\le {d}_{0}\right)}^{n}\left[\frac{{S}_{i}f({b}_{iu},{b}_{id}{,b}_{ju}{,b}_{jd})}{{\sum }_{k\in \left({d}_{ki}\le {d}_{0}\right)}^{m}\left({D}_{k}f({b}_{iu},{b}_{id}{,b}_{ju}{,b}_{jd})\right)} \right]$$

Equation (7) replaces the broadband communication strengths function $f({b}_{i}{b}_{j})$ in Eq. (4) with a step-wise weight function $f({b}_{iu},{b}_{id}{,b}_{ju}{,b}_{jd})$. The communication strengths in Eq. (7) takes into account the upload and download speed of patients and doctors, as well as the video conferencing software’s speed requirement.

### Data processing

We will outline the steps taken to process the data and calculate telehealth accessibility in this section:

- (1) We established a 15-mile buffer zone around Cook County and extracted Census Block data within both the county and the buffer for analysis. The final results will focus on Cook County itself, as shown in Fig. 1a.
- (2) For supply data, we extracted the locations of individual doctors within Cook County and the surrounding 15-mile buffer zone and aggregated them into Census Blocks. We used Census block centroids to represent doctor’s locations. The aggregation was performed because Internet speed data is only available at the block level.
- (3) We computed a large OD matrix based on OSRM and OpenStreetMap road networks in this study. This OD matrix consisted of more than 27 billion (166,980 * 166,980= 27,882,320,400) records.
- (4) According to Zoom’s video conference broadband speed requirements, we assigned weights to the broadband speed as outlined in Table 1 (see also Eqs. (5), (6)). Consequently, a weights table was generated for each Census Block pair, which was joined back to the OD matrix based on the origin and destination computed in the previous step.
- We applied all the processed data to Equation (7) to calculate telehealth accessibility.

**Fig. 2:**
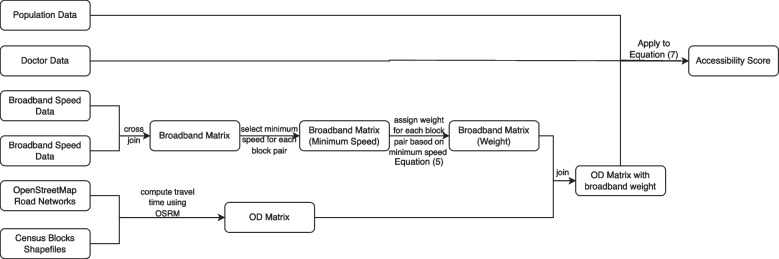
Data processing flow chart

The above steps are summarized in the following chart (Fig. 2).

## Results and discussions

### Results

**Fig. 3:**
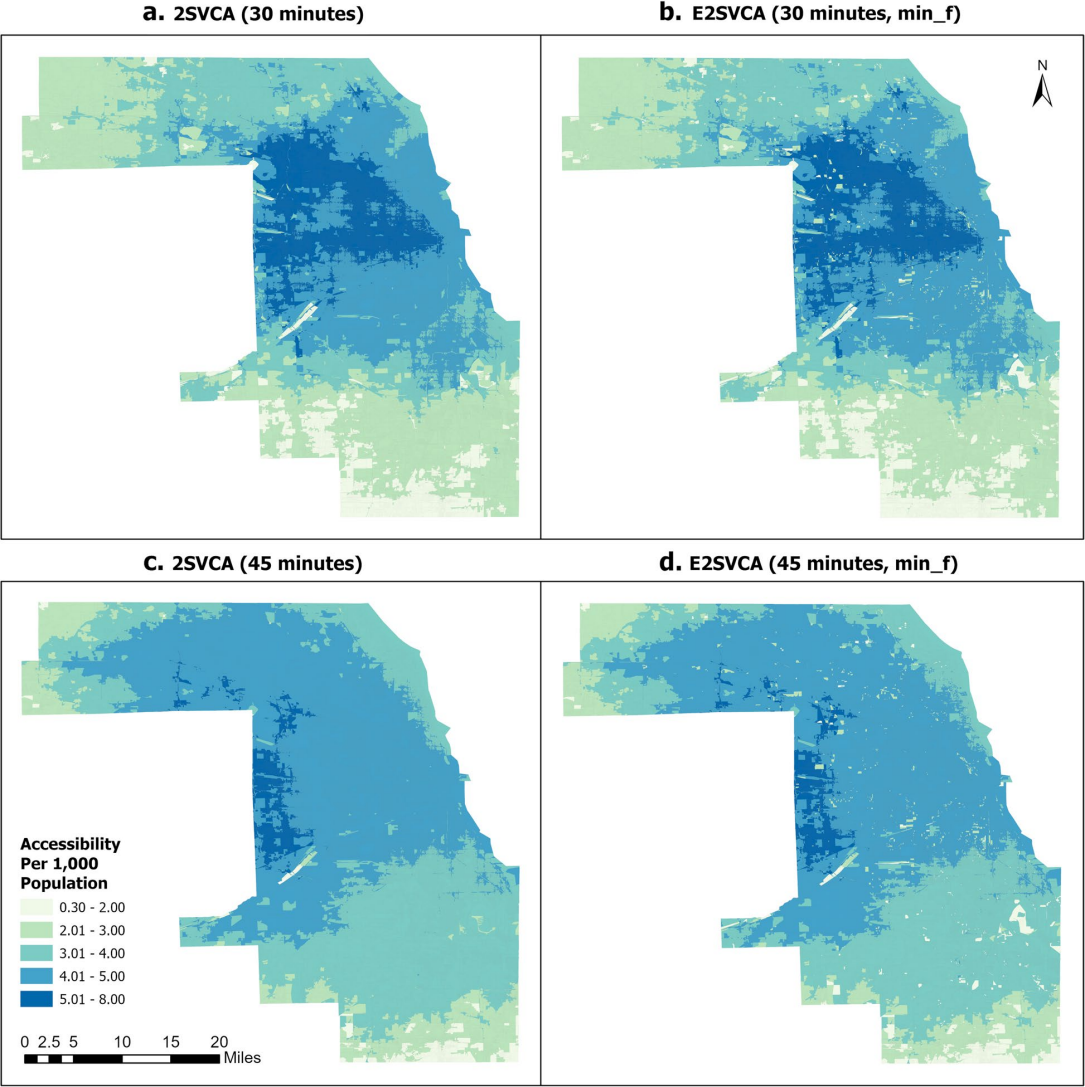
2SVCA and E2SVCA Results

The telehealth accessibility results for the 30-min catchment size per 1,000 population (without normalization applied) are displayed in Fig. 3a and b. Figure 3a depicts the spatial distribution of telehealth accessibility scores obtained from the 2SVCA method in which each Census Block has at least one Internet service provider, causing $f({b}_{i}{b}_{j})=1$ in all pairs of Census Blocks. As a result, the model is essentially the same as the conventional 2SFCA model. Figure 3b demonstrates the results of the spatial distribution of the E2SVCA accessibility score using the minimum of the most frequently available broadband speeds (min_f) in a Census Block. Both methods exhibit a similar overall pattern in Cook County. The south edge and southwest area of Cook County have very poor accessibility scores (0–2.00). The majority of the south and northwest parts of Cook County have relatively low telehealth accessibility scores (2.01–3.00). Almost all of the middle-level (3.01–4.00) accessibility areas are located in the north of Cook County except for a small part in the south. A significant number of Census Blocks situated in central Cook County have better accessibility (4.01–5.00) than the middle level. The high (5.01–8.00) accessibility regions spread outward from Downtown Chicago and toward the west and northwest.

Figure 3c and d illustrate the accessibility results for the 45-min catchment size. Compared to Fig. 3a and b, the 45-min results are generally smoother, with the highest accessibility category concentrated on the central west edge of Cook County. This smoothing effect is likely due to the larger catchment size. The overall patterns of 2SVCA and E2SVCA results for the 45-min catchment size are similar, but the E2SVCA exhibits many Census Blocks with lower accessibility scores than 2SVCA (lighter color “speckles” dispersed in the darker background in Fig. 3d). This is also true in Fig. 3b. Thus, we can conclude that both 2SVCA and E2SVCA can reveal telehealth accessibility, but the 2SVCA model visually overestimated the telehealth accessibility in some regions. (More details will be discussed in the next section).

### Different broadband speed variables

The FCC’s broadband speed dataset contains multiple records in many areas due to the presence of multiple service providers offering Internet services in the same Census Block. In the 2SVCA model, the average download and upload speed were used to represent the Internet speed of a Census Block. However, assuming that a Census Block has a few service providers with high network speeds and many service providers with low network speeds, the average network speed may not be a reliable representation of the Census Block’s Internet speed.

For example, let’s consider Census Block 170,318,391,002,021, which has ten service providers offering upload speeds of 0.5, 1.3, 2.0, 3.0, 3.0, 3.0, 20.0, 35.0, 200.0, and 1000.0. The average speed for this Census Block is 126.8. However, if we exclude the extreme values of 200.0 and 1000.0, the average speed drops to 6.8. It is quite apparent that the average value does not accurately represent the Internet speed for that particular Census Block.

In order to determine the most appropriate metric for measuring network speed in a Census Block and compare it to the 2SVCA model, this study introduces three new metrics: min_a, max_f, and min_f.

- (1) min_a refers to the minimum available network speed in the Census Block. In cases where there are multiple Internet service providers in a Census Block, the lowest available network speed will be selected as the min_a.
- (2) min_f represents the most frequent speed in the Census Block if there is only one most frequent speed.
- (3) max_f is used when a Census Block has more than one most frequent Internet speed. In such cases, the maximum (max_f) and minimum (min_f) of the most frequent speeds are selected separately. However, if only one most frequent speed is available, then max_f and min_f will be the same (see case (2) above).

For example, in Census Block 170,310,102,011,004, there are 10 different available Internet download speeds (2, 3, 18, 25, 25, 35, 100, 500, 1000, 1000). However, the two most frequent speeds are 1000Mbps and 25Mbps (they both occur twice), resulting in max_f = 1000 Mbps, min_f = 25 Mbps, and min_a = 2Mbps. Hereafter we only use max_f and min_f; it is important to note that they are not the minimum and maximum of all available speeds (which would be 2 and 1000 in this case), only the minimum and maximum of the most frequent speeds (thus the “_f” suffix).

**Fig. 4:**
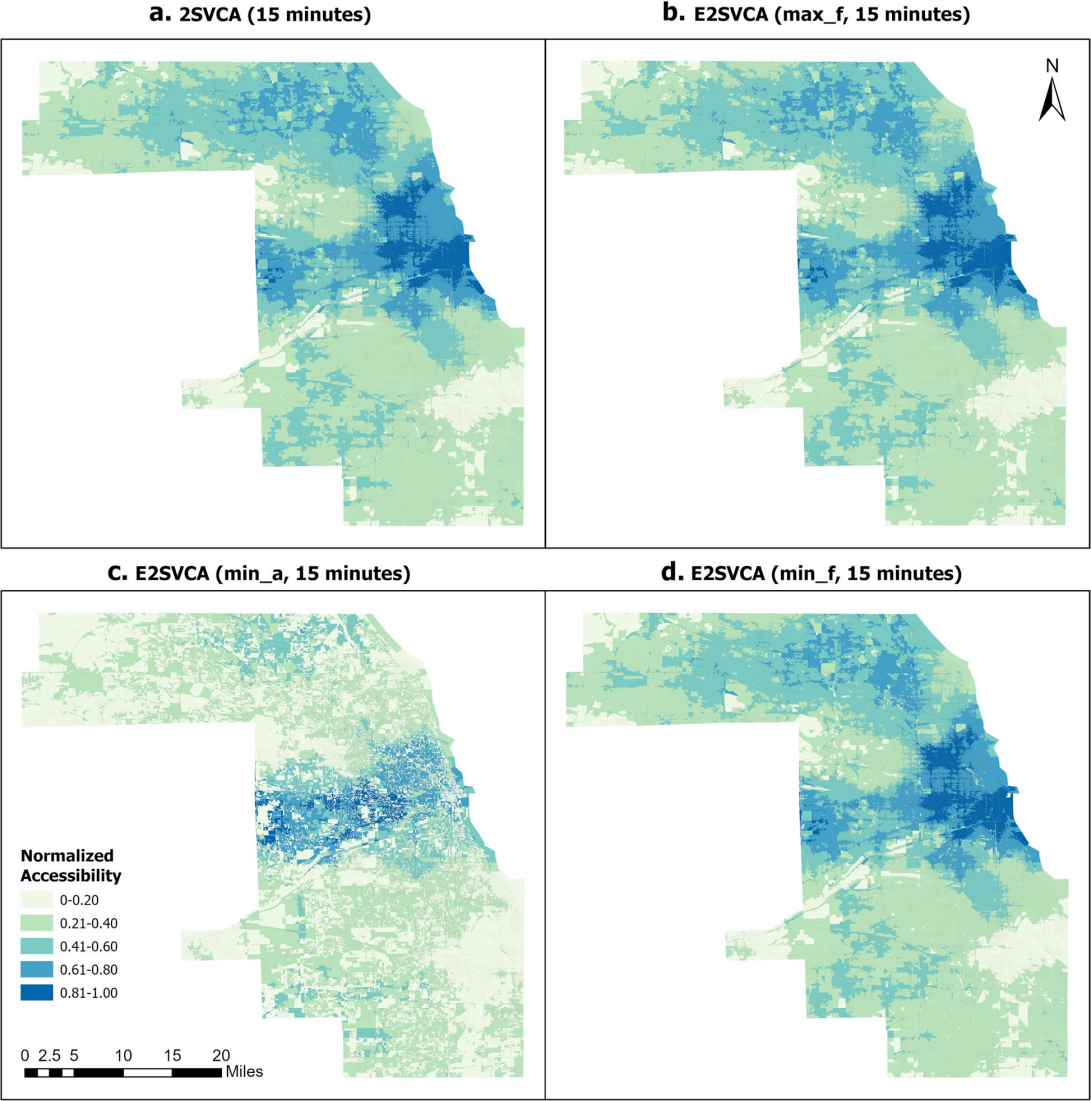
Normalized 15 min 2SVCA and E2SVCA Results

**Fig. 5:**
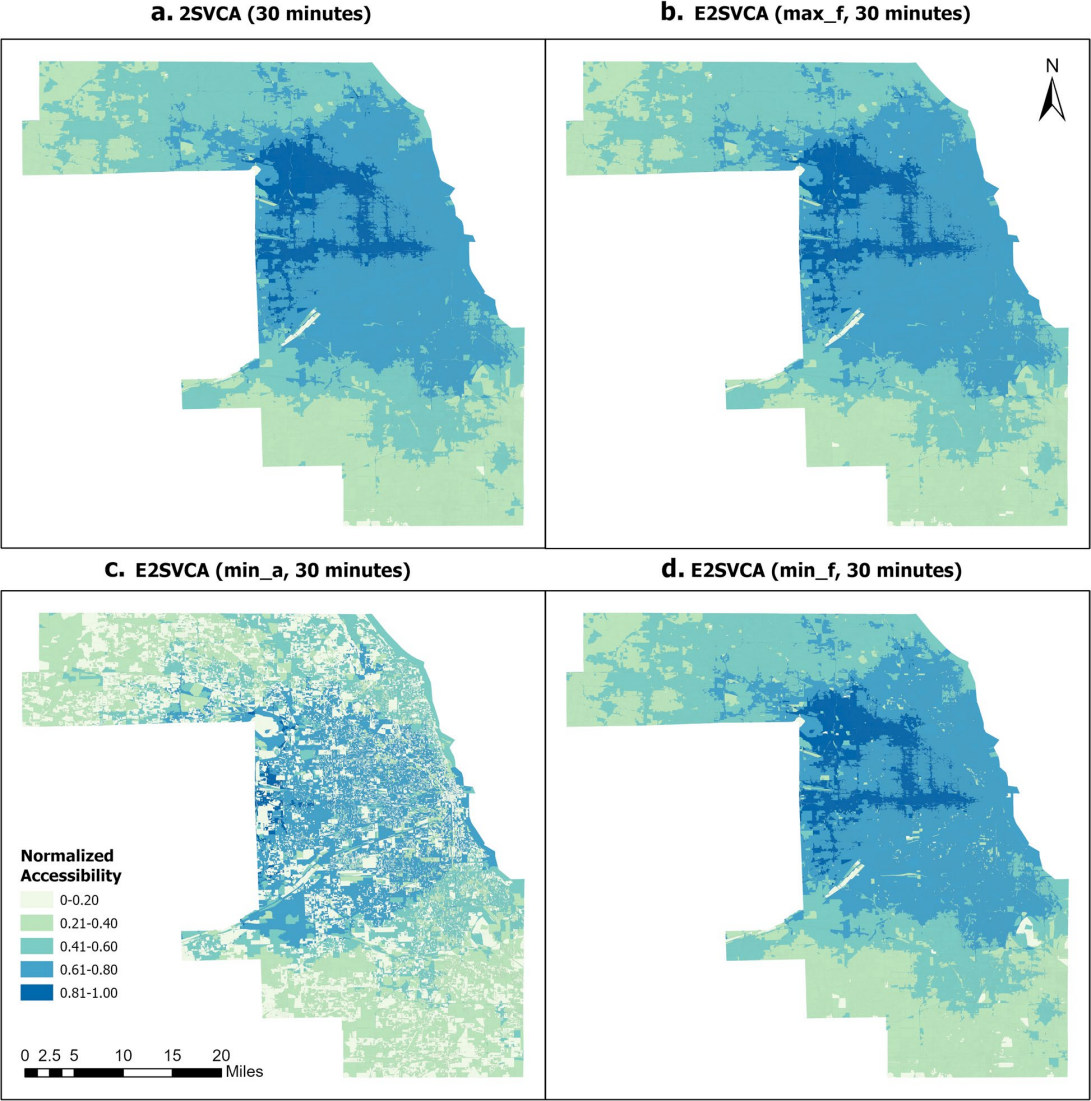
Normalized 30 min 2SVCA and E2SVCA Results

**Fig. 6:**
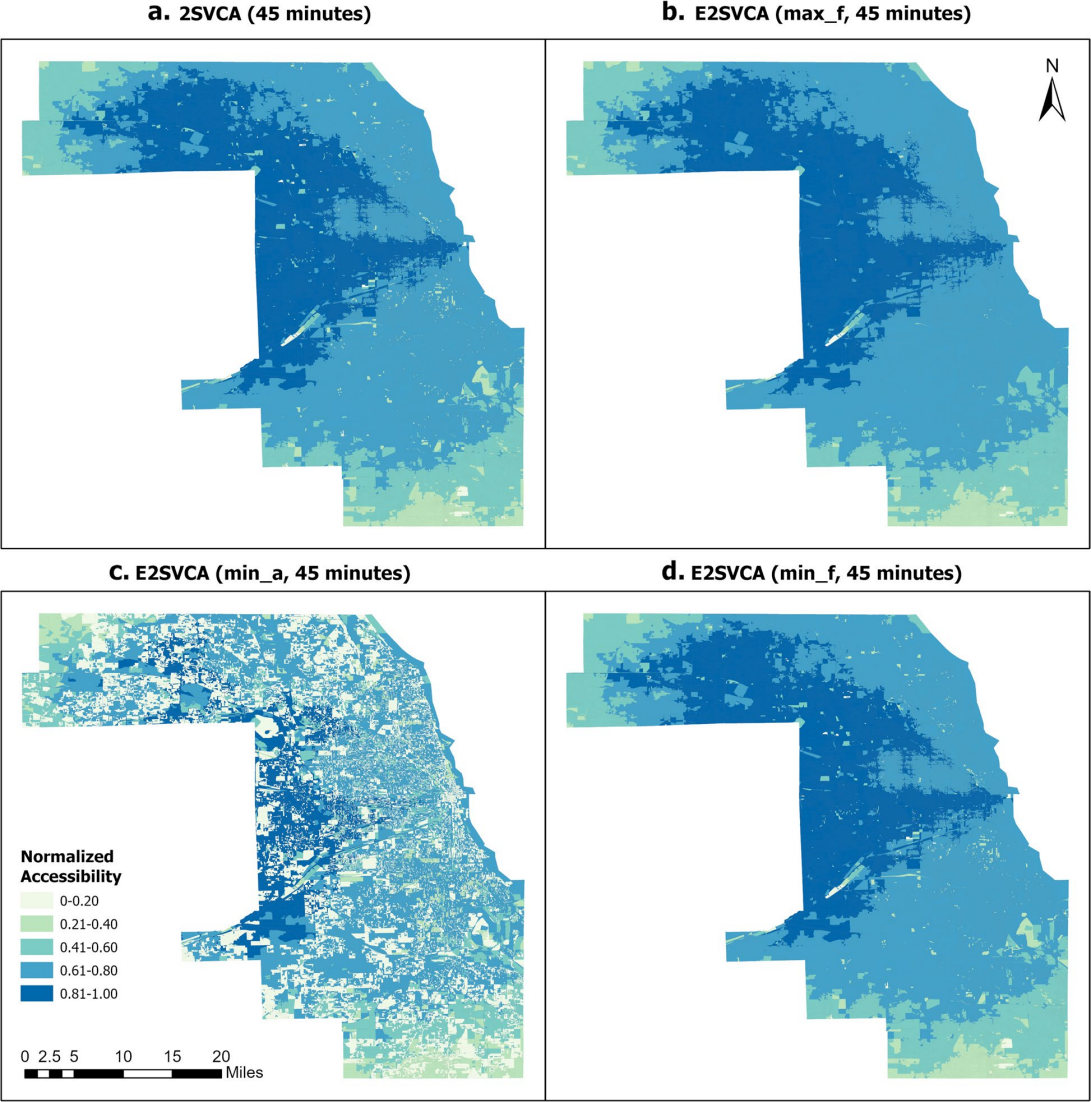
Normalized 45 min 2SVCA and E2SVCA Results

Figures 4, 5 and 6 illustrate the normalized results of the three indicators introduced above which are utilized in the E2SVCA model for various catchment sizes. To facilitate differentiation and expression, we divided them into four groups (Table 2):

**Table 2:** Figure groups

Groups	Method	Metric	Figures
Group 1	2SVCA		Figures 4a, 5a, 6a
Group 2	E2SVCA	max_f	Figures 4b, 5b, 6b
Group 3	E2SVCA	min_a	Figures 4c, 5c, 6c
Group 4	E2SVCA	min_f	Figures 4d, 5d, 6d

To ensure a more precise comparison, we also included the results of 2SVCA (Group 1). In our analysis, all the accessibility scores are normalized to the range of 0–1 and divided into the same five classes for better comparison.

The results below illustrate that Fig. 4a and b exhibit similar patterns of telehealth accessibility, as do Figs. 5a and b, and 6a, b. The only difference between the two models is the weight function used in the calculation process. In the 2SVCA model, the weight is 1 (i.e., $f({b}_{i}{b}_{j})=1$ in Eq. (4) since all Census Blocks in the study area have Internet). On the other hand, the E2SVCA model employs one of the three weight metrics to compute the weight for each Census Block. If we utilize max_f as the Internet speed indicator for weight calculation but obtain similar patterns for both the 2SVCA model and E2SVCA model, it indicates that most of the weights used in the E2SVCA model are equal to 1. Considering that 2SVCA is the best case of telehealth accessibility without considering the Internet bandwidth constraints, we can conclude that using max_f as the Internet speed indicator provides the optimal case of telehealth care accessibility.

Comparing the results of E2SVCA using min_a (Group 3, Figs. 4c, 5c, 6c) with the 2SVCA results (Group 1, Figs. 4a, 5a, 6a), the most notable characteristic of the minimum available (min_a) Internet speed results is the discontinuity of the accessibility scores and significantly lower accessibility scores. The results show that a large number of Census Blocks have very low accessibility scores, surrounded by high accessibility areas. The minimum available (min_a) Internet speed presents the worst scenario of patients’ telehealth care accessibility at the Census Block level.

After comparing the outcomes of 2SVCA (Group 1, Figs. 4a, 5a, 6a) with those obtained from E2SVCA using min_f (Group 4, Figs. 4d, 5d, 6d), it is clear that most of the Census Blocks in the study area have the same accessibility categories. However, the E2SVCA model indicates a higher number of Census Blocks with low accessibility scores compared to the 2SVCA model. These findings suggest that the 2SVCA model tend to overestimate the accessibility results in some regions.

### Different catchment sizes

In our previous study, we determined that a 60-min catchment size is sufficient to reach almost anywhere in Cook County (Shao & Luo, 2022). Therefore, we will focus on discussing the accessibility of catchment with shorter travel times, specifically the 15-min, 30-min, and 45-min catchments.

As we increase the catchment size from 15 to 45 min, we observe similar overall patterns of change from one catchment size to the next (keeping everything else the same, e.g., from Figs. 4a to 5a, from Figs. 4b to 5b). Regions with high and mid-high accessibility scores expanded, and high accessibility regions shifted from the central and eastern parts of the county to the west and northwest. With a catchment size of 15 min, patients have limited options for seeking telehealth care within that restricted range, and the high telehealth accessibility is concentrated in the Downtown Chicago region, where a large number of doctors are located. When the catchment size increases to 30 min, high accessibility (0.81–1.0) clusters move to the west part of Cook County, and the number of low (0–0.20) accessibility Census Blocks significantly decreases compared to the 15-min catchment. The high (0.81–1.00) accessibility covers a significant portion of Cook County, except for the north and south parts. The results reveal that both the south part of Cook County and the northwest of Cook County experienced low telehealth care accessibility. As the catchment increases to 45 min, the pattern changes dramatically, and high (0.81–1.0) accessibility areas cover almost all of west Cook County, extending to the north of Cook County. However, the south and northwest edges of Cook County still have low telehealth care accessibility. We have included data from a 15-mile buffer around Cook County in all our calculations, so this is unlikely due to the edge effect.

As the catchment size increases, the pattern of change for Group 3 (min_a) differs from that of the other groups (Group 1, Group 2, Group 4). In particular, Group 3 has a significant number of areas with lower scores than the other three groups. Many of the areas with low accessibility scores remain low regardless of changes in catchment size. This is mainly due to the extremely low Internet speeds (min_a) in Group 3.

### Quantitative comparison between the 2SVCA and the E2SVCA (30 minutes)

**Fig. 7:**
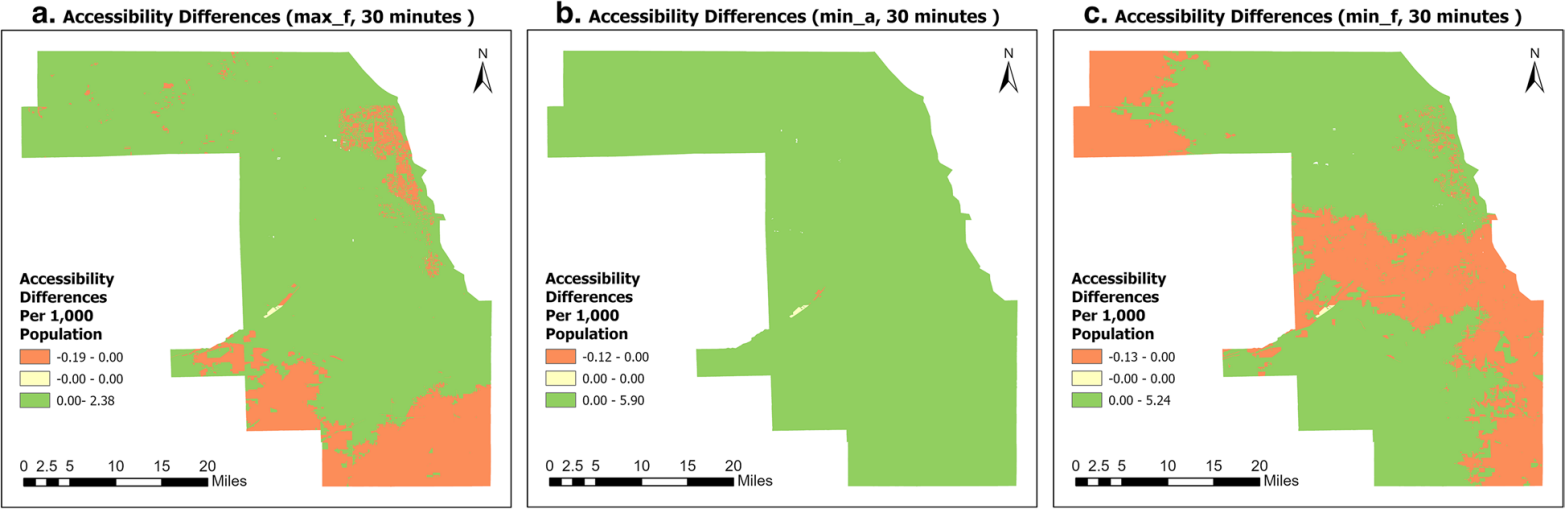
Differences between 2SVCA and E2SVCA

In addition to examine various different Internet speed metrics, we also generated difference maps that compare the 30-min catchment accessibility (per 1,000 population) results of the 2SVCA model and E2SVCA model. The results were obtained by subtracting the E2SVCA outcomes from the 2SVCA outcomes (no normalization applied). To better illustrate the findings on the maps, we classified the results into three classes (as shown in Fig. 7): less than 0 (orange), equal to 0 (yellow), and greater than 0 (green).

The orange color (2SVCA < E2SVCA) on the map indicates that the 2SVCA model has underestimated accessibility in those areas, while the green color (2SVCA > E2SVCA) indicates that 2SVCA has overestimated accessibility. Figure 7a reveals that the underestimation areas extend northward along Lake Michigan from Downtown Chicago, with a few underestimated Census Blocks scattered in the north of Cook County. Another cluster of underestimations is located in the south and southwest of Cook County. In Fig. 7b, it is demonstrated that 2SVCA overestimates accessibility in almost all blocks in Cook County, except for a few Census Blocks in the west. Figure 7c shows that one of the underestimation clusters extends from Downtown Chicago to the north of the county, similar to the pattern in Fig. 7a. Two more underestimated clusters are located on the county's west edge in the north, and on the county's east edge, which also stretching from east to west. In conclusion, we can see that 2SVCA results overestimate accessibility in the majority of the areas in all three different metrics cases while underestimating in other areas compared to E2SVCA.

Tables 3, 4, and 5 provide summary of the statistics for the results presented above. Table 3 shows that 26 Census Blocks have an accessibility score of 0, indicating that for both methods the accessibility scores are identical. For the remaining blocks, there are more positive differences than negative differences in all three metrics, indicating that the 2SVCA model generally produced higher accessibility scores than E2SVCA. Therefore, we can conclude that the 2SVCA method overestimated accessibility in most Census Blocks compared to E2SVCA. Table 4 displays the distribution of the population across different categories. By combining the information from Tables 3 and 4, we find that the majority of the population (83.47%, 100%, 62.01% for these three metrics) lives in Census Blocks where the 2SVCA method overestimates accessibility scores.

**Table 3:** Number and percentage of Census Blocks with differences in the accessibility scores computed from 2SVCA and E2SVCA methods

Differences	Less than 0	0	Greater than 0
max_f	13,420 (13.55%)	26 (0.03%)	85,586 (86.42%)
min_a	13 (0.01%)	26 (0.03%)	98,993 (99.96%)
min_f	38,942 (39.32%)	26 (0.03%)	60,064 (60.65%)

**Table 4:** Number and percentage of Census Blocks with differences in the population computed from 2SVCA and E2SVCA methods

Differences	Less than 0	0	Greater than 0
max_f	858,834(16.53%)	0	4,335,841(83.47%)
min_a	0	0	5,194,675(100.00%)
min_f	1,973,585(37.99%)	0	3,221,090(62.01%)

**Table 5:** Summary statistics of accessibility scores (per 1,000 population)

Methods	Mean	Std	Min	Max
2SVCA	4.14	1.02	0	6.48
E2SVCA (max_f)	4.13	1.01	0	6.470
E2SVCA (min_a)	2.02	1.20	0	4.57
E2SVCA (min_f)	4.12	1.04	0	6.48

Table 5 presents the summary statistics of the accessibility scores for 2SVCA and E2SVCA. The results of 2SVCA and E2SVCA (max_f) are quite similar in terms of their range, average value, and standard deviations. However, the results of E2SVCA (min_a) differ significantly from those of 2SVCA. In 2SVCA, the accessibility scores range from 0 to 6.48, while in E2SVCA (min_a), they range from 0 to 4.57, with an average score of 2.02, which is half of the average score in 2SVCA (4.14). Furthermore, E2SVCA (min_a) has a higher standard deviation (1.20) than 2SVCA (1.02), indicating that the scores in E2SVCA (min_a) are more spread out. On the other hand, E2SVCA (min_f) has a slightly smaller average score than 2SVCA, but a sliglty larger standard deviation. The E2SVCA (min_a) results, in particular, show that the model produces lower scores with greater variability than 2SVCA, highlighting the importance of using the appropriate metric for measuring accessibility.

## Limitations and future work

There are a variety of telehealth care methods, such as remote patient monitoring, video meetings, etc., but this study only focuses on video communication between patients and doctors. Moreover, the study only has access to Internet speed data at the Census Block level from available service providers, not the actual usage by users. Therefore, this study only measures potential virtual accessibility. To simplify the video calling models, the study uses the minimum download and upload Internet speed and assumes that all communication between patients and doctors is conducted via Zoom, which holds the majority market share in the United States. However, video calling quality is influenced by various factors in the real world, such as the devices (Ethernet or WIFI), the router location and also the network stability. Since the study lacks such data, these factors were not considered. The study also faced data consistency issue, as it used data from the 2010 Census that comprises 166,980 Census Blocks, while there are 166,986 Census Blocks in the FCC broadband data. We ignored the minor difference between these two datasets. Additionally, the 15-mile buffer zone to mitigate the edge effect may need to be reconsidered as the transportation networks have significantly improved.

Furthermore, this study assumed that all individuals in the study area would seek health care remotely and that all providers would offer telehealth care services. However, a more precise telehealth care accessibility result could be obtained with a health care facilities dataset containing actual telehealth care providers. This study simplified the communication model by assuming that the minimum Internet speed (for both upload and download) between patients and doctors is the key factor affecting video quality. If additional data were available, it would be interesting to incorporate other network metrics, such as network latency into the model to determine video conference quality. Additionally, telehealth care services that occur during peak times of network demand may also impact the video quality, and considering alternative times for telehealth care may be important to ensure smooth communication during heavy network demand.

## Conclusion

This paper presents the Enhanced Two-Step Virtual Catchment Area (E2SVCA) model for evaluating access to telehealth, building on the previous Two-Step Virtual Catchment Area (2SVCA) method. The previous 2SVCA method oversimplified the model by considering only a binary case: with or without Internet access and using the average Internet speed as the speed of the Census Block, which did not capture the full picture of telehealth accessibility, as not all areas with Internet have sufficient speed to meet telehealth needs. The new E2SVCA model takes into account different bandwidth categories and replaces the binary broadband strength joint function with a step-wise function that more accurately reflects the requirements of telehealth video conferencing. We have explored several broadband speed indicators (min_a, min_f, max_f) at the Census Blocks level, and found that min_a is the best option for accessing the worst-case scenario of telehealth care accessibility, as it evaluates the accessibility using the minimum available Internet speed. min_f and max_f represent the most common cases of telehealth accessibility with the former focusing on the common case and the latter emphasizing optimal accessibility. All three indicators suggest that the 2SVCA model overestimates telehealth care accessibility, whereas the E2SVCA model provides more accurate measurements. This finding expands the current literature on telehealth accessibility evaluation and can help policy makers allocate health care resources more effectively to improve healthcare equality and patient outcomes.

## Data Availability

The data that support the findings of this study will be available on GitHub (https://github.com/861/Enhanced-Two-Step-Virtual-Catchment-Area-Model-to-Measure-Telehealth-Accessibility).
